# Interoception and borderline personality disorder in female adolescents with restrictive eating disorders

**DOI:** 10.1007/s40519-026-01889-7

**Published:** 2026-07-21

**Authors:** Anna Riva, Carlo Panarella, Gabriele Arienti, Simona Di Guardo, Eleonora Brasola, Renata Nacinovich, Andrea E. Cavanna

**Affiliations:** 1 Department of Child Neuropsychiatry, Fondazione IRCCS San Gerardo Dei Tintori, 20900 Monza, Italy; 2 School of Medicine and Surgery, Università degli Studi di Milano – Bicocca, Via Cadore 48, 20900 Monza, Italy; 3 Department of Neuropsychiatry, BSMHFT and School of Medical Sciences, College of Medicine and Health, University of Birmingham, Birmingham, UK; 4 School of Health and Life Sciences, Aston Institute of Health and Neurodevelopment, Aston University, Birmingham, UK; 5 Sobell Department of Motor Neuroscience and Movement Disorders, Institute of Neurology and University College London, London, UK

**Keywords:** Adolescents, Restrictive eating disorders, Interoception, Borderline personality disorder

## Abstract

**Purpose:**

Interoception has been reported to be impaired in patients with restrictive eating disorders (REDs), while its role in patients with borderline personality disorder (BPD) is more controversial. We set out to conduct a clinical study to explore the relationship between interoception, eating disorders, and BPD.

**Methods:**

We recruited a sample of 75 female adolescents affected by REDs, of whom 21 received a diagnosis of comorbid BPD. In addition to collecting demographic and clinical data, we administered measures of interoceptive awareness (MAIA-2), the scale for eating symptomatology (EDI-3), and alexithymia (TAS-20) to all patients.

**Results:**

Patients with REDs and comorbid BPD reported lower scores on the MAIA-2 Not-Distracting (p = 0.046) and Trusting (p = 0.023) subscales, reflecting decreased focus and reliance on bodily sensations of pain or discomfort compared to patients with REDs only. Lower scores on the MAIA-2 Trusting subscale were associated with higher scores on all EDI-3 subscales and on the total TAS-20 scale (p = 0.005), whereas lower scores on the MAIA-2 Not-Worrying subscale were associated with higher scores on the EDI-3 Interpersonal Problems composite subscale (p = 0.049), and higher scores on the MAIA-2 Noticing subscale were associated with higher scores on the EDI-3 Affective Problems composite subscale (p = 0.003).

**Conclusions:**

Interoceptive dysfunction appears to be a key vulnerability factor for eating disorder psychopathology in female adolescents with REDs, with comorbid BPD potentially further exacerbating these impairments. Targeting interoceptive processes may represent a promising therapeutic approach to improve emotion regulation and clinical outcomes, particularly in patients with co-occurring BPD, and warrants further investigation in this clinical population.

*Level III* Evidence obtained from cohort or case–control analytic studies.

## Introduction

Interoception is defined as "the process by which the nervous system senses, interprets, and integrates signals originating from within the body, providing a moment-to-moment mapping of the body's internal landscape at both conscious and unconscious levels" [1]. Traditionally, interoception was considered to be related to internally originating signals, such as visceral signals, heartbeat, and respiratory rate. However, more recent conceptualizations of interoception include higher-order representations of skin and body temperature, pain, itch, and sensory touch [2, 3]. Interoception plays a crucial role in maintaining internal homeostasis, motivating behavior, and guiding decision-making. For example, the perception of a compromised nutritional state in the body drives food-seeking and consumption behaviors [4]. Converging evidence from neuroanatomical and neuroimaging studies identified the anterior insular cortex as a key neural substrate for deficits in interoception [2, 5, 6], which have been reported across multiple psychiatric disorders [1, 7, 8], including eating disorders (EDs) [9–11] and borderline personality disorder (BPD) [12, 13].

EDs are severe psychiatric conditions associated with substantial medical [14–16] and psychological burden [17, 18]. Psychiatric comorbidities affect approximately 50–80% of patients with EDs [19–21]. Among these, personality disorders are frequently reported, with prevalence estimates ranging from 27 to 93% [22]. BPD is among the most extensively studied personality disorders in relation to EDs and has been associated with greater psychopathological severity, emotion dysregulation, interpersonal difficulties, unstable self-image, impulsivity, and increased rates of self-harm and suicidal behaviors [23–26]. Conversely, individuals with BPD show elevated rates of lifetime eating disorder diagnoses, suggesting a substantial overlap between these clinical conditions [27, 28]. Difficulties in emotion regulation have been proposed as a transdiagnostic mechanism underlying both EDs and BPD, potentially contributing to their frequent co-occurrence [29, 30].

Interoceptive deficits have long been recognized as a key feature of EDs [31]. Bruch (1982) [32] first documented disturbances in the perception and interpretation of bodily signals in patients with anorexia nervosa, which were subsequently conceptualized as impairments in interoception [33]. More recent evidence suggests that interoceptive dysfunction extends beyond anorexia nervosa and may represent a transdiagnostic characteristic across eating disorder diagnoses [10]. Notably, a comprehensive systematic review on interoception and disordered eating reported interoceptive impairments across different eating disorder diagnoses and interoceptive modalities, thereby supporting the hypothesis that interoceptive dysfunction may represent a core feature of eating psychopathology [10]. Altered interoception has also been documented in individuals at risk of developing EDs [3] and appears to persist following clinical recovery [34, 35], further supporting its role as a core psychopathological dimension of EDs [36].

The relationship between interoception and BPD has received comparatively less attention and remains incompletely understood. Although early studies failed to identify significant alterations in interoceptive processing among individuals with BPD [37], more recent investigations have reported impairments across several interoceptive dimensions [12, 13]. The ability to perceive and regulate emotions is closely linked to interoception [38]. A growing body of evidence suggests that abnormalities in interoceptive processing may contribute to core features of BPD, including emotional instability, disturbances in self-experience, and interpersonal dysfunction [12]. Recent evidence further suggests that alterations in emotional processing and regulation, which are characteristic of BPD, may be associated with deficits in the representation of bodily signals [39, 40].

Despite growing evidence supporting the role of interoceptive dysfunction in EDs and BPD, studies directly examining interoceptive processes in individuals with comorbid EDs and BPD remain scarce, with available evidence largely limited to small clinical samples and preliminary investigations. To date, research has predominantly examined interoceptive dysfunction in EDs and BPD separately [10, 41]. Moreover, studies of interoception in EDs have frequently relied on mixed diagnostic samples, making limited contributions to our understanding of disorder-specific interoceptive profiles [10, 41]. Consequently, little is known about interoceptive functioning in adolescents with restrictive eating disorders (REDs) including anorexia nervosa, atypical anorexia nervosa, avoidant/restrictive food intake disorder (ARFID), and other non-specified EDs with restrictive characteristics [19], who also present with comorbid BPD. Investigating this population may help clarify whether the association between interoceptive dysfunction and borderline personality pathology also extends to restrictive eating psychopathology, an area that has only recently become the focus of empirical investigation [42].

Accordingly, the present study aimed to assess interoceptive functioning in a clinical sample of female adolescents with REDs, comparing patients with and without comorbid BPD. We hypothesized that the presence of BPD would be associated with greater impairment across interoceptive dimensions. A further aim of the present study was to evaluate the relationship between different interoceptive dimensions/BPD and the clinical phenomenology of the eating disorder, including alexithymia in the context of REDs.

## Materials and methods

### Study design and participants

A total of 75 female adolescents aged 12–17, of whom 21 with BPD, received a DSM-5-TR-validated diagnosis of REDs following a specialist assessment at the Department of Child Neuropsychiatry, Fondazione IRCCS San Gerardo dei Tintori, Monza, Italy. The semi-structured screening interview for personality disorders—SCID-5-PD [43, 44] was administered to all subjects to validate the diagnosis of comorbid BPD. Implementation of the SCID-5-PD to the diagnostic evaluation of adolescents was encouraged following slight modifications to the wording of questions [45]. Patients with EDs secondary to medical conditions, severe psychotic disorders, personality disorders different from BPD, intellectual disability, and insufficient proficiency in Italian language were excluded from the present study.

All adolescents and their parents were provided with detailed information about the purpose of the study, and written informed consent was obtained from the participants’ parents.

### Measures

An ad hoc questionnaire was designed to systematically collect demographic (age) and clinical characteristics (premorbid body mass index-BMI, BMI, difference between BMI at diagnosis and premorbid BMI (delta BMI) and REDs diagnosis.

The Multidimensional Assessment of Interoceptive Awareness-2 (MAIA-2) assesses multidimensional aspects of self-reported interoception and includes 37 items scored on a six-point Likert scale (from 0, never to 5, always) [46]. Higher scores indicate higher levels of self-reported interoception. The questionnaire includes eight subscales: Noticing (“awareness of uncomfortable, comfortable, and neutral body sensations”), Not-Distracting (“tendency not to ignore or distract oneself from sensations of pain or discomfort”), Not-Worrying (“tendency not to worry or experience emotional distress with sensations of pain or discomfort”), Attention Regulation (“ability to sustain and control attention to body sensations”), Emotional Awareness (“awareness of the connection between body sensations and emotional states”), Self-Regulation (“ability to regulate distress by attention to body sensations”), Body Listening (“active listening to the body for insight”), and Trusting (“experience of one’s body as safe and trustworthy”). Cronbach’s alpha values for the eight subscales range from 0.64 to 0.83.

The Eating Disorder Inventory-3 (EDI-3) is a self-report tool measuring psychological traits relevant to individuals with EDs. It consists of 91 items organized into 12 primary scales: three eating disorder-specific scales (Drive for Thinness, Bulimia, Body Dissatisfaction) and nine general psychological scales (Low Self-Esteem, Personal Alienation, Interpersonal Insecurity, Interpersonal Alienation, Interoceptive Deficits, Emotional Dysregulation, Perfectionism, Asceticism, and Maturity Fears) that are highly relevant to, but not specific for, EDs. The primary scales yield six composite scores: one eating disorder-specific risk score (Eating Disorder Risk Composite), and five scales that tap into general integrative psychological constructs (Ineffectiveness, Interpersonal Problems, Affective Problems, Overcontrol, and Global Psychological Maladjustment). The EDI-3 has been field-tested in females with EDs in a wide (13–53 years) age range. The Italian version of the EDI-3 demonstrated high internal reliability coefficients (0.80–0.90) and test–retest reliability coefficients (0.93–0.98) [47]. EDI-3 scores greater than 85 are considered pathological, whereas scores between 70 and 85 are borderline.

The Toronto Alexithymia Scale (TAS-20) is a 5-point Likert type self-report questionnaire consisting of 20 items that assess the core features of alexithymia, namely difficulties in identifying (DIF) and describing (DDF) feelings, and lack of focus on internal emotional experiences (EOT). The presence of alexithymia traits is predicted by a total score > 61. The Italian version of the TAS-20 showed good internal consistency (Cronbach’s alpha values of 0.75 and 0.82 in healthy and clinical groups, respectively) and high test–retest reliability over two weeks (r = 0.86) [48]. Confirmatory factor analysis revealed the same factor structure as the original English version and adequate internal consistency of the subscales, with alpha coefficients equal or greater than 0.70 [48].

### Data analysis

Since data were not normally distributed, continuous variables were presented as median and skewness coefficients, whereas categorical variables were presented as absolute or percent frequencies. Comparisons of psychological features among the two groups were conducted through univariate analyses using the nonparametric Mood’s median test. Chi-squared and Fisher’s exact tests were performed to compare categorical variables. Both MAIA subscale scores and presence of BPD were entered as independent variables into a multiple regression analysis model with EDI-3 composite scores and TAS-20 total score as dependent variables. The level of significance was set at p < 0.05. Statistical analyses were performed using the SPSS 28.0 package (IBM Corp. Released 2019. IBM SPSS Statistics for Windows, Version 26.0. Armonk, NY: IBM Corp).

## Results

The socio-demographic and clinical characteristics of the REDs groups with and without BPD are summarized in Table 1. The two study groups were homogeneous for age, premorbid body mass index (BMI), BMI at diagnosis, difference between BMI at diagnosis and premorbid BMI (delta BMI), and REDs phenotype.

**Table 1:** Socio-demographic and clinical features in female adolescents with REDs

	REDs with BPD (N = 21)	REDs without BPD (N = 54)	p value
Age, median (skewness)	17.0 (−0.06)	16.0 (−0.35)	0.106
Premorbid BMI, median (skewness)	19.8 (1.73)	20.0 (0.68)	0.943
BMI at diagnosis, median (skewness)	15.5 (0.68)	16.6 (1.15)	0.658
Delta BMI, median (skewness)	4.7 (0.55)	3.6 (0.37)	0.558
AN*, N (%)*	19 (90.5)	40 (74.1)	
ARFID*, N (%)*	0 (0.0)	3 (5.6)	0.258
OSFED*, N (%)*	2 (9.5)	11 (20.4)	

Group comparisons were performed using Mood’s median test for continuous variables and Fisher’s exact tests for categorical variables, as appropriate

*N* number, *AN* Anorexia Nervosa, *ARFID* Avoidant/Restrictive Food Intake Disorder, *OSFED* other non-specified eating disorders with restrictive characteristics, *BMI* Body Mass Index, *BPD* Borderline Personality Disorder, *REDs* Restrictive Eating Disorders. * p < 0.05, ** p < 0.01, *** p < 0.001

The comparative analysis of TAS-20, EDI-3, and MAIA-2 scores between the two groups is detailed in Table 2. Patients with REDs and comorbid BPD reported lower scores on the MAIA-2 Not-Distracting (p = 0.046) and Trusting (p = 0.023) subscales, all EDI-3 composite scores (except for the Overcontrol composite scale), and TAS-20 total scores (p = 0.003) and subscores (DIF: p = 0.014; DDF: p = 0.007; EOT: p = 0.401), compared to patients with REDs only.

**Table 2:** Toronto alexithymia Scale-20 (TAS-20), eating disorder inventory-3 (EDI-3) and multidimensional assessment of interoceptive awareness-2 (MAIA-2) scores in female adolescents with REDs

	REDs with BPD (N = 21)		REDs without BPD (N = 54)		
	*Median*	*Skewness*	*Median*	*Skewness*	*p-value*
TAS-20	70.0	−0.90	61.0	−0.20	0.003**
TAS-20 DIF	28.0	−0.67	22.0	−0.17	0.014**
TAS-20 DDF	21.0	−0.32	18.0	−0.32	0.007**
TAS-20 EOT	22.0	−0.00	20.0	0.14	0.401
EDI-3 EDRC	90.0	−1.56	76.5	−1.00	0.005**
EDI-3 IC	89.0	−1.72	85.5	−1.00	0.05*
EDI-3 IPC	92.0	−1.42	74.0	−0.73	< 0.001***
EDI-3 APC	93.0	−1.44	78.0	−0.67	0.006**
EDI-3 OC	82.0	−1.12	69.0	−0.81	0.154
EDI-3 GPMC	91.5	−1.23	83.0	−1.16	0.019**
MAIA-2 noticing	2.8	−0.25	2.6	−0.24	0.868
MAIA-2 not-distracting	1.8	1.03	2.5	0.44	0.046*
MAIA-2 not-worrying	2.0	0.09	2.1	0.06	0.415
MAIA-2 attention regulation	1.9	0.17	2.0	0.32	0.549
MAIA-2 emotional awareness	2.4	0.25	2.8	−0.29	0.602
MAIA-2 self-regulation	0.8	0.68	1.3	0.84	0.194
MAIA-2 body listening	1.0	0.45	1.0	0.78	0.325
MAIA-2 Trusting	1.0	0.72	1.7	0.49	0.023*

*N* number, *BPD* Borderline Personality Disorder, *TAS-20* Toronto Alexithymia Scale-20, *DDF* difficulty describing feelings, *DIF* difficulty identifying feelings, *EOT* externally-oriented thinking, *EDI-3* Eating Disorder Inventory-3, *EDRC* Eating Disorder Risk Composite, *IC* Ineffectiveness, *IPC* Interpersonal Problems, *APC* Affective Problems, *OC* Overcontrol, *GPMC* Global Psychological Maladjustment, *MAIA-2* Multidimensional Assessment of Interoceptive Awareness-2. * p < 0.05, ** p < 0.01, *** p < 0.001

The multiple linear regression analysis model (Table 3) with EDI-3 composite scores and TAS-20 total score as dependent variables and both MAIA subscale scores and presence of BPD as independent variables (predictors) reached statistical significance (F = 6.185, p < 0.001). Lower MAIA-2 Trusting scores were associated with higher scores across all EDI-3 subscales and the TAS-20 total score (p = 0.005), whereas lower MAIA-2 Not-Worrying scores were associated with higher EDI-3 Interpersonal Problems scores (p = 0.049), and higher MAIA-2 Noticing scores were associated with higher EDI-3 Affective Problems scores (p = 0.003).

**Table 3:** Multiple linear regression analyses with MAIA-2 scores and the presence of BPD as independent variables and EDI-3 scores and TAS-20 scores as dependent variables

	EDI-3 EDRC		EDI-3 IC		EDI-3 IPC		EDI-3 APC		EDI-3 OC		EDI-3 GPMC		TAS-20	
Predictor variables	Beta	p-value	Beta	p-value	Beta	p-value	Beta	p-value	Beta	p-value	Beta	p-value	Beta	p-value
Age	0.756	0.612	0.015	0.901	3.121	0.139	2.097	0.189	-0.504	0.822	0.736	0.678	1.215	0.210
MAIA-2 noticing	2.220	0.265	0.103	0.389	1.919	0.491	6.589	0.003**	2.393	0.423	2.676	0.234	2.544	0.051
MAIA-2 not-distracting	1.000	0.663	0.016	0.905	−4.789	0.141	−0.948	0.697	1.317	0.703	−1.854	0.479	−1.489	0.317
MAIA-2 not-worrying	−2.449	0.250	−0.175	0.161	−5.947	0.049*	−1.309	0.560	−1.988	0.532	−3.811	0.125	−2.361	0.088
MAIA-2 attention regulation	0.800	0.751	0.172	0.242	3.010	0.396	0.670	0.803	2.857	0.452	3.228	0.278	1.019	0.532
MAIA-2 emotional awareness	2.097	0.240	0.077	0.560	−2.039	0.413	2.219	0.242	4.381	0.104	3.356	0.104	−1.355	0.240
MAIA-2 Self-regulation	1.441	0.518	−0.009	0.945	2.950	0.346	0.489	0.836	−2.091	0.533	−0.292	0.906	0.693	0.630
MAIA-2 Body listening	−1.361	0.589	−0.225	0.141	−2.169	0.539	−2.919	0.277	−3.703	0.329	−3.910	0.166	−0.462	0.776
MAIA-2 Trusting	−9.873	< 0.001***	−0.433	0.005**	−7.605	0.011*	−8.130	< 0.001***	−6.568	0.038**	−7.799	0.001***	−3.877	0.005**
BPD	5.349	0.265	.0168	0.186	10.207	0.131	8.538	0.096	1.061	0.882	6.068	0.280	3.331	0.282
R^2^	0.539		0.419		0.424		0.565		0.278		0.518		0.432	

*TAS-20* Toronto Alexithymia Scale-20, *EDI-3* Eating Disorder Inventory-3, *EDRC* Eating Disorder Risk Composite, *IC* Ineffectiveness, *IPC* Interpersonal Problems, *APC* Affective Problems, *OC* Overcontrol, *GPMC* Global Psychological Maladjustment, *MAIA-2* Multidimensional Assessment of Interoceptive Awareness-2, *BPD* Borderline Personality Disorder. * p < 0.05, ** p < 0.01, *** p < 0.001

## Discussion

The present study builds on the growing field of research on REDs by focusing on the relationship between interoception and eating disorder severity, as well as the role that interoceptive dysfunction may play in the context of a common psychiatric comorbidity such as BPD. BPD has been consistently linked to greater severity of eating disorder psychopathology, higher clinical impairment, and poorer overall outcomes in individuals with EDs, as shown in recent meta-analytic and clinical evidence [49–51].

However, despite growing evidence supporting the role of interoception in EDs, to our knowledge no studies have specifically investigated interoceptive functioning in patients with REDs and comorbid BPD to date.

We compared self-reported levels of interoception between two groups of female adolescents with REDs: 21 patients with comorbid BPD and 54 with REDs only. The two groups were homogeneous in terms of both BMI (previous *versus* current) and REDs phenotype; however patients with REDs and comorbid BPD reported greater severity in both eating disorder symptomatology (EDI-3 scores) and alexithymia (TAS-20 scores). These findings are in line with a growing body of literature reporting similar results [49–51].

Moreover, female adolescents with REDs and comorbid BPD reported significantly lower scores on the MAIA-2 Not-Distracting and Trusting subscales, suggesting that the presence of BPD further exacerbates aspects of interoceptive impairment. Difficulties in interpreting and regulating bodily signals may reflect a broader inability to regulate emotions, a defining characteristic of BPD. Additionally, impairments in recognizing and understanding internal emotional states may contribute to affective instability [24]. Therefore, interoceptive deficits in BPD may be explained through the framework of emotional dysregulation, a core feature of BPD [39, 40].

The Not-Distracting subscale measures an individual's tendency not to ignore their bodily sensations, with lower score indicating that subjects tend to divert attention from unpleasant bodily sensations or dissociate from their interoceptive experiences. Impairment in this aspect of interoception could be consistent with psychopathological processes specific to both REDs and BPD, for several reasons. First, BPD is strongly associated with dissociation: many people with this disorder report feeling disconnected from their bodies, especially during episodes of intense emotional distress [52]. Diverting attention from bodily sensations is considered a maladaptive coping strategy used to avoid facing intolerable emotions (e.g., anger, emptiness, emotional pain). BPD is characterized by a cycle of hypersensitivity and hypercontrol: when emotional pain becomes too intense, the subject may react by disconnecting from their body [53]. Thus, lower scores on the Not-Distracting subscale would indicate that subjects with BPD consciously avoid focusing on bodily sensations, consistent with their tendency toward dissociation and experiential avoidance [54]. Furthermore, ignoring physical sensations of discomfort was most strongly connected to symptoms of restraint and fear of losing control over eating [55].

The Trusting subscale measures an individual's trust in their bodily sensations, therefore lower scores on this subscale indicate distrust in own body signals and their reliability. Alterations in this aspect of interoception are consistent with psychological characteristics of BPD. One of the key aspects of BPD is identity instability, which often manifests as a distorted perception of the body and difficulty integrating bodily sensations into one's self-experience. Many subjects with BPD have a history of early trauma or abuse, which may result in a conflicted relationship with the body [56]. In cases of childhood trauma, it is common for individuals to develop distrust in bodily signals because the body has been a source of pain or because they have learned to dissociate from it as a form of self-protection. Some subjects with BPD are aware of their bodily sensations (contrary to what the Not-Distracting domain suggests) but perceive them as chaotic, unmanageable, and dangerous. For example, a patient might feel their heartbeat accelerate during a panic attack, but instead of interpreting it as a momentary stress signal, they might perceive it as a catastrophic or unmanageable occurrence. Thus, lower score on the Trusting component of interoception would indicate that subjects with BPD do not trust their body as a reliable guide, reflecting their identity instability, their use of dissociation as a coping strategy, and their difficulty regulating emotions through bodily signals. Moreover, in previous studies trust in bodily perceptions have been related to both general and specific psychopathology of EDs [11, 57].

In summary, our results suggest that adolescents with REDs and BPD have a more severe impairment in interoception than adolescents with REDs only. Their interoceptive impairment is characterized by avoiding and distrusting their bodily sensations. This is reflected in lower scores on the MAIA-2 Not-Distracting and Trusting subscales, which are most directly linked to dissociation and negative body perception.

The scores on the different MAIA-2 subscales and the presence of comorbid BPD were included as independent variables in separate multiple linear regression models to determine which factors might be associated with eating disorder symptoms and alexithymia. Our findings suggest that lower scores on the MAIA-2 Trusting subscale were associated with higher scores across all EDI-3 subscales as well as the total TAS-20 score, whereas lower MAIA-2 Not-Worrying scores were associated with higher scores on the EDI-3 Interpersonal Problems subscale. Additionally, higher scores on the MAIA-2 Noticing subscale predicted higher scores on the Affective Problems scale of the EDI-3.

Interestingly, there was no evidence that the presence of comorbid BPD contributes to either eating disorder symptoms or alexithymia in the present sample. While this finding may appear counterintuitive in light of previous evidence linking BPD traits to greater emotional dysregulation and overall clinical severity in EDs, it is consistent with a transdiagnostic interpretation of psychopathology. In particular, interoceptive dysfunction has been increasingly identified as a core mechanism underlying both eating disorder symptoms and emotional processing difficulties across clinical populations, including BPD [1, 58].

Within this framework, the present findings suggest that interoceptive dimensions, especially trust in own bodily sensations, may represent a more proximal and explanatory factor than diagnostic comorbidity. Notably, lower scores on the MAIA-2 Trusting subscale were consistently associated with greater eating disorder psychopathology and alexithymia, suggesting that difficulties in trusting bodily sensations may underlie both affective and eating-related symptoms. Accordingly, the lack of an independent effect of BPD may indicate that its contribution to clinical severity is partially mediated or overshadowed by interoceptive dysfunction, which appears to capture more directly the variance in psychopathological outcomes. Finally, the clinical homogeneity of the sample, composed exclusively of female adolescents with REDs, and the developmental instability of borderline personality traits may have further limited the incremental explanatory value of BPD in the regression models.

In summary, the results of our regression analysis suggest that, in female adolescents with REDs, different dimensions of interoception (Noticing, Not-Worrying, and Trusting), but not comorbid BPD, act as risk factors for the development of specific eating disorder symptoms and alexithymia.

## Strength and limits

The strengths of this study lie in its design, originality of focus, and use of multidimensional psychometric instruments. Limitations include the relatively small sample size and limited generalizability in consideration of potential referral bias, as all participants were recruited from a tertiary center specializing in EDs.

## Conclusions

The results of our study highlight the importance of a thorough assessment of interoceptive skills in patients with REDs, especially in the context of comorbid BPD. The evidence emerging from our work suggests that interoceptive rehabilitation might become a therapeutic area for patients with EDs associated with BPD. Specifically, psychotherapeutic techniques aimed at improving body-emotion awareness could represent effective interventions to enhance emotional regulation and prevent self-harming behaviors. In patients with REDs and BPD, a therapeutic approach focused on bodily reconnection and trust in own somatic sensations could be more beneficial than simply improving interoceptive awareness.

In conclusion, our findings are consistent with the hypothesis that the interoceptive impairment associated with BPD could represent a vulnerability that increases the risk of dysfunctional eating disorder symptoms. By addressing these difficulties, treating clinicians could not only improve emotion regulation, but also enhance treatment response. Based on our preliminary findings, the connection between interoception, emotional dysregulation, and eating behavior should be the focus of further research. In particular, it would be helpful to explore the role of interoception and BPD for the risk of self-harm behaviors in adolescents with REDs, with the ultimate goal of developing targeted treatment interventions to improve clinical outcomes in patients with EDs and BPD.

## What is already known on this subject?

Interoceptive deficits are recognized as a key feature of EDs and may represent a transdiagnostic factor across different diagnoses. However, possible variations in interoceptive impairment related to specific personality traits have not yet been thoroughly explored. This issue is particularly relevant considering that BPD is the most common comorbid personality disorder in individuals with REDs, with estimated prevalence rates around 10%.

## What this study adds

Our findings suggest that the presence of BPD further exacerbates interoceptive impairments in adolescents with REDs. Interoceptive deficits associated with BPD may represent a vulnerability factor that increases the risk for more severe and dysfunctional eating disorder symptoms. These results indicate that targeting interoceptive processes could represent a promising therapeutic approach for individuals with EDs and BPD.

## Data Availability

The data that support the findings of this study are available from the corresponding author upon reasonable request. Approval was obtained from the local ethics committee. Approval was obtained from the local ethics committee.
